# Pinostrobin mitigates neurodegeneration through an up-regulation of antioxidants and GDNF in a rat model of Parkinson’s disease

**DOI:** 10.12688/f1000research.134891.2

**Published:** 2023-10-27

**Authors:** Ratchaniporn Kongsui, Tichanon Promsrisuk, Lars Klimaschewski, Napatr Sriraksa, Jinatta Jittiwat, Sitthisak Thongrong

**Affiliations:** 1 Division of Physiology, School of Medical Sciences, University of Phayao, Mueang Phayao District, Phayao, 56000, Thailand; 2 Division of Neuroanatomy, Department of Anatomy Histology and Embryology, Innsbruck Medical University, Innsbruck, 6020, Austria; 3 Faculty of Medicine, Mahasarakham University, Maha Sarakham, Maha Sarakham, 44000, Thailand; 4 Division of Anatomy, School of Medical Sciences, University of Phayao, Mueang Phayao District, Phayao, 56000, Thailand

**Keywords:** Pinostrobin, Parkinson’s disease, Oxidative stress, Boesenbergia rotunda

## Abstract

**Background**: One of the most common neurodegenerative diseases is Parkinson’s disease (PD); PD is characterized by a reduction of neurons containing dopamine in the substantia nigra (SN), which leads to a lack of dopamine (DA) in nigrostriatal pathways, resulting in motor function disorders. Oxidative stress is considered as one of the etiologies involved in dopaminergic neuronal loss. Thus, we aimed to investigate the neuroprotective effects of pinostrobin (PB), a bioflavonoid extracted from *Boesenbergia rotunda* with antioxidative activity in PD.

**Methods**: Rats were treated with 40 mg/kg of PB for seven consecutive days before and after 1-methyl-4-phenyl-1,2,3,6-tetrahydropyridine (MPTP)-induced PD. After completing the experiment, the brains including SN and striatum were used for histological studies and biochemical assays.

**Results**: PB treatment demonstrated a reduction of free radicals in the SN as indicated by significantly decreased MDA levels, whereas the antioxidative enzymes (SOD and GSH) were significantly increased. Furthermore, PB treatment significantly increased glial cell line-derived neurotrophic factor (GDNF) immunolabelling which has neurotrophic and neuroprotective effects on the survival of dopaminergic neurons. Furthermore, PB treatment was shown to protect CA1 and CA3 neurons in the hippocampus and dopaminergic neurons in the SN. DA levels in the SN were increased after PB treatment, leading to the improvement of motor function of PD rats.

**Conclusions**: These results imply that PB prevents MPTP-induced neurotoxicity via its antioxidant activities and increases GDNF levels, which may contribute to the therapeutic strategy for PD.

## Introduction

One of the most common neurodegenerative diseases is Parkinson’s disease (PD), which has a high prevalence worldwide. ^1^ PD is characterized by a reduction of neurons containing dopamine in the substantia nigra pars compacta (SNpc), which leads to a lack of dopamine (DA) in the nigrostriatal pathways. DA insufficiency in the striatum leads to movement disorders including muscle rigidity, postural imbalance, resting tremor and bradykinesia. ^2^^,^^3^ Currently, PD cannot be cured, but symptomatic treatment with L-3,4-dihydroxyphenylalanine (levodopa, L-DOPA) and DA receptor agonists exists. However, the effects of these medications are not long-lasting and they unsuccessful in preventing the loss of dopamine neurons in the SNpc. ^4^^,^^5^ So far, accumulative studies have shown that dopaminergic neuronal loss in the SNpc is a progressive process; however, its exact mechanisms remain poorly understood. ^6^^–^^8^ Even though the pathogenesis of PD is still unclear, oxidative stress has been suggested as a possible mechanism of dopaminergic neuronal death in the SNpc. ^9^^,^^10^ 1-methyl-4-phenyl-1,2,3,6-tetrahydropyridine (MPTP) is a neurotoxin which is widely used to target dopaminergic neuronal death in animal models of PD. ^11^^,^^12^ The active metabolite of MPTP, 1-methyl-4-phenyl pyridinium (MPP ^+^), is selectively transported into dopaminergic neurons and causes mitochondrial dysfunction through a massive accumulation of free radicals such as reactive oxygen species (ROS), resulting in neuronal apoptosis. ^13^^,^^14^

Pinostrobin (PB, 5-hydroxy-7-methoxyflavanone), a bioactive flavonoid, is found in the rhizomes of *Boesenbergia rotunda* (fingerroot or Krachai) in Thailand. ^15^ PB has various bioactivities and pharmacological effects, including antioxidative, anti-peptic ^16^ anti-inflammatory, analgesic, ^17^ anticancer, ^18^ and neuroprotective activities. ^19^ Our previous studies have shown that treatment of PB at the doses of 20 and 40 mg/kg improved peripheral nerve regeneration and functional recovery after injury in rats through attenuation of oxidative stress. ^20^ Recently, the antioxidative effects of PB against neurotoxin-induced dopaminergic neurons loss have been demonstrated in zebra fish and in SH-SY5Y cells. ^21^ However, a single animal study cannot fully elucidate the effects of PB on PD; therefore, the neuroprotective and therapeutic effects of PB must be further investigated. Current pharmacological therapeutics can only increase DA level and improve motor function of PD patients but are unsuccessful at preventing neurodegeneration. Thus, this research was set up to explore possible neuroprotective effects of PB in a rodent model of PD.

## Methods

### Preparation of PB

PB was prepared from *B. rotunda* rhizomes as previously described in our work (20). Briefly, fresh rhizomes were ground then dried for an ethanolic extract. The extract was resuspended in methanol to obtain a pale yellow solid. The structural analysis of PB was performed by 13C- and 1H-NMR spectroscopy (Bruker Avance DRX500 Spectrometer, USA) and by nuclear magnetic resonance (NMR) spectra for comparison with published data.

### Animal procedures and MPTP injection

Eighteen male Wistar rats (200-220 g) were purchased from Nomura Siam International Co., Ltd (Thailand). All rats were acclimatized for 1 week in a room with 12 h light-dark cycle and constant temperature (25±2°C) with food and water access *ad libitum.* All animal procedures were approved by the Animal Ethics Committee of University of Phayao, Thailand, with approval number 640104028. The animals were randomly divided into three groups: control group, MPTP group and MPTP+40 mg/kg PB group. MPTP (Tokyo Chemical Industry Co., Ltd. Cat#M2690, Japan) was freshly diluted in 0.9 % sterile normal saline for the intraperitoneal injection (i.p.) to induce PD at a dose of 20 mg/kg once per day for five consecutive days. In the MPTP+40 mg/kg PB group, the animals were additionally treated with PB dissolved in 0.5% of carboxymethylcellulose sodium (Sigma-Aldrich, USA) once daily by oral gavage for seven consecutive days before and after MPTP injection.

### Ladder rung walking test

**Figure 1.**
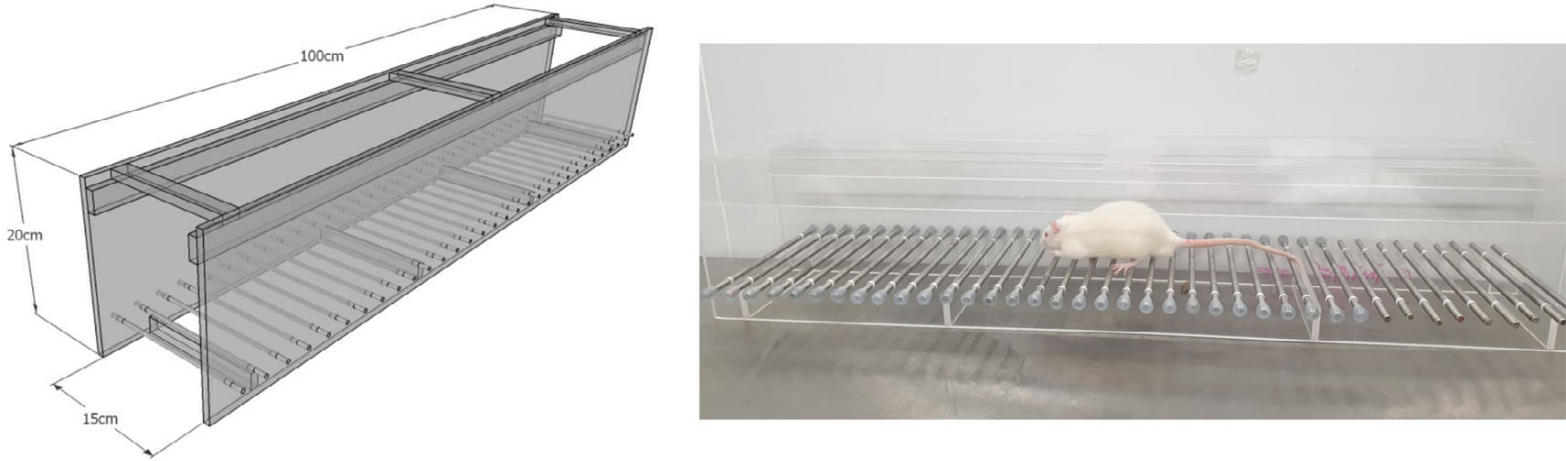
Ladder rung walking apparatus for testing the motor deficit induced by 1-methyl-4-phenyl-1,2,3,6-tetrahydropyridine.

A horizontal ladder rung walking apparatus was used to test the motor coordination between forelimbs and hindlimbs as previously described by Metz and Whishaw. ^22^ All animals spontaneously walked from a beginning point to a goal point along a horizontal rung consisting of 100 × 20 cm clear acrylic walls. The metal rungs created a walking space with a minimum distance of 1 cm between rungs ( Figure 1). All animals were trained to cross the ladder for three days before i.p. injection of MPTP. Seven days after MPTP injection, all animals were required to walk across the ladder for assessing motor function recovery. All animals were tested three times per session and the spending time to cross the rungs was recorded.

### Tissue preparation for biochemical assays

After completing the behavioral experiment, all animals were i.p. injected with thiopental sodium at a dose of 70 mg/kg followed by ice cold 0.9% sterile normal saline perfusion through the heart. The right hemisphere of the midbrain including substantia nigra was dissected and collected for biochemical assays. The tissues were homogenized in a homogenate buffer containing 50 mM Tris-HCl (pH 7.4), 150 mM NaCl, 1% Triton X-100, and protease inhibitor and then centrifuged at 10,000 rpm at 4°C for 15 min. The protein concentrations were determined by the method of Lowry. Bovine serum albumin (BSA) (Cat# 9048-46-8, Sigma-Aldrich, USA) was used as a standard. ^23^

### Malondialdehyde (MDA) assay

MDA assay was performed according to Luangaram and co-workers. ^24^ Briefly, 150 μL of tissue supernatant was mixed with chemical solutions as described in the protocol. The mixed solution was incubated at room temperature (RT) for 10 min. After that, 500 μL of 0.6% thiobarbituric acid (TBA) was added to the mixture and then boiled for 30 min followed by centrifugation at 2,800 g for 20 min. Thiobarbituric acid-reactive substances (TBARS) were detected at 532 nm using the Cytation5 microplate reader (USA). A standard curve was produced using appropriate concentrations of 1,1.3,3-tetraethoxypropane (0.3–10 μmol/L). The concentration of MDA in each tissue sample was normalized to protein content. All MDA assays were done three times.

### Glutathione (GSH) assay

The oxidative stress response was determined by the measurement of reduced GSH content following Polycarp and colleagues’s protocol. ^25^ In brief, 1 mM reduced GSH (Cat# 70-18-8, Sigma-Aldrich, USA) was diluted in 0.1 N HCl to make serial dilutions of 0.2, 0.4, 0.6 and 0.8 mM GSH for a standard curve. The reaction was started by adding each serial dilution or tissue sample to the phosphate buffer pH 7.6 and 1 mM 5,5-dithio-bis-(2-nitrobenzoic acid) (DTNB) (Cat# 69-78-3, Merk, USA). Thereafter, the mixture was incubated for 5 min in the dark at RT. Each sample mixture was read at 412 nm using a Cytation5 microplate reader. GSH concentration in tissue sample was normalized to protein content, and the experiments were repeated three times.

### Superoxide dismutase (SOD) assay

SOD in response to oxidative stress was detected using a SOD Assay Kit (Cat# 19169, Sigma-Aldrich, USA) following the manufacturer's protocol. The SOD concentration was normalized to protein content, and the experiments were repeated three times.

### Measurement of DA

DA expression was detected using a Dopamine ELISA Kit (Cat# E-EL-0046, Elabscience, USA) following the manufacturer's instructions. The results were normalized to protein content, and the experiments were performed in triplicates.

### Nissl staining for determination of the density of hippocampal neurons

The left hemisphere of brain tissue samples was fixed in 4% paraformaldehyde in phosphate buffered saline (PBS) pH 7.4 at 4°C for 48 h, then cryoprotection in 30% sucrose for 48 h. The brains were coronally cut at a thickness of 20 μm using a cryostat (CM1950, Leica Microsystems, Inc., Germany). Brain sections were used for Nissl staining using a cresyl violet solution (Cat# 105235, Sigma-Aldrich; Merck KGaA, USA) to determine the neuronal density in the hippocampal sub-fields including cornu ammonis 1 (CA1) and cornu ammonis 3 (CA3). The 40× magnification images of the CA1 and CA3 regions (at stereotaxic co-ordinates 4.0-4.2 mm posterior to the bregma) were chosen for measuring the neuronal density using the NIS Elements imaging software version 5 (Nikon Corporation). The experiments were done three times, and the data were shown as % of control.

### Immunohistochemistry

To determine dopaminergic neurons in the SNpc, brain coronal sections at stereotaxic co-ordinates 4.6-4.8 mm behind the bregma were incubated with tyrosine hydroxylase (TH) antibodies. Positive GDNF immunolabelling in the striatum (at 0.5 mm anterior to the bregma) was detected using GDNF antibodies. Sections were free-floating-blocked with 10% normal goat serum and 0.3% Triton X-100 in PBS at RT for 90 min. TH primary antibody (1:500, Cat# ab6211, Abcam, UK) or GDNF primary antibody (1:500, Cat# ab244211, Abcam, UK) were applied overnight at RT followed by biotin-SP-conjugated donkey anti-rabbit secondary antibody (1 : 500, Code: 711-065-152 Jackson Immunoresearch, USA) or biotin-SP-conjugated donkey anti-mouse secondary antibody (1 : 500, Code: 715-065-150 Jackson Immunoresearch, USA) for 2 h at RT. After that, sections were incubated for 1 h in 0.1% extravidin peroxidase (1:1000, Cat# E2886, Sigma-Aldrich, USA) then detected by 3,3′-diaminobenzidine (DAB). The images were examined at 40× magnification by NIS Elements imaging software version 5 (Nikon). TH-positive neurons or GDNF positive immunolabelling were counted and expressed as a percentage of control. The experiments were done three times on each animal.

### Statistical analysis

Results were analyzed by one-way ANOVA followed by a Tukey's *post hoc* using GraphPad Prism version 9 (GraphPad Software, Inc., USA) and presented as Means±SEM for motor functional tests, histology, and immunohistochemical studies, Mean±SD for biochemical assays. Statistical significance was considered when p-values were lower than 0.05 (p<0.05).

## Results

### PB ameliorates the MPTP-induced motor function deficit

**Figure 2.**
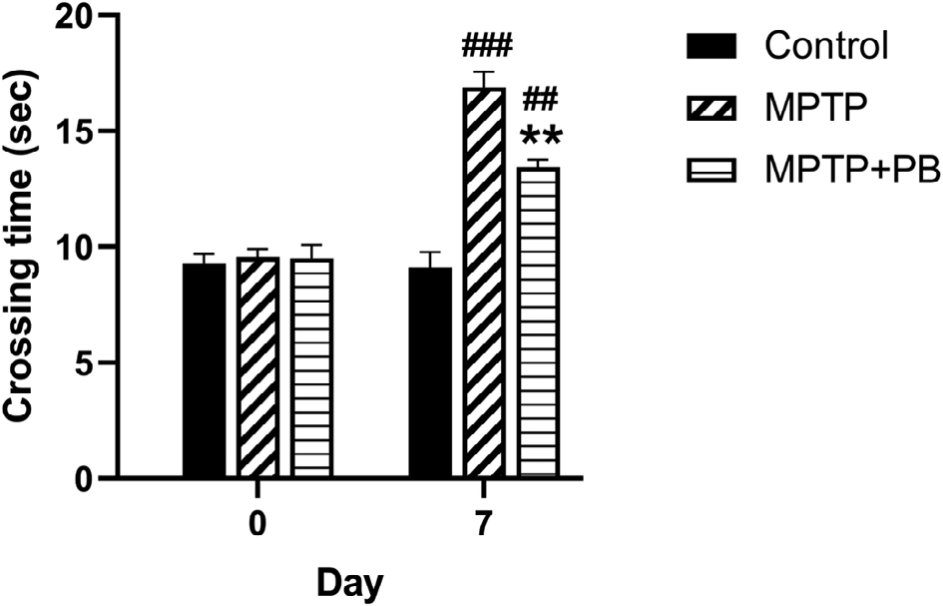
MPTP-induced motor lesions were observed on day seven after injection. Pinostrobin-treated animals exhibited less time spent on the ladder when compared to animals in the 1-methyl-4-phenyl-1,2,3,6-tetrahydropyridine only group. Data were reported as mean±SEM (n=6), one-way ANOVA. ^##^p<0.01, and ^###^p<0.001 *versus* control group; **p<0.01 *versus* MPTP group.

To evaluate the effect of PB on MPTP-induced motor functional deficit, all animals were assigned to walk across the ladder rung apparatus. Prior to MPTP injection at baseline, there were no significant differences in crossing time between all animal groups (Control: 9.27±0.41 s *versus* MPTP: 9.56±0.35 s *versus* MPTP+PB: 9.50±0.57 s, Figure 2). Seven days after MPTP injection, the MPTP and MPTP+PB groups showed a significantly increased time spent across the ladder rungs when compared to the control group (Control: 9.11±0.78 s *versus* MPTP: 16.89±0.88 s *versus* MPTP+PB: 13.44±0.16 s). The data indicated that administration of MPTP negatively affects motor functions; however, treatment with PB significantly improved motor coordination, resulting in reduced crossing times as compared to the MPTP group (p<0.01).

### Effects of PB on oxidative stress, antioxidant activities and DA expressions

**Figure 3.**
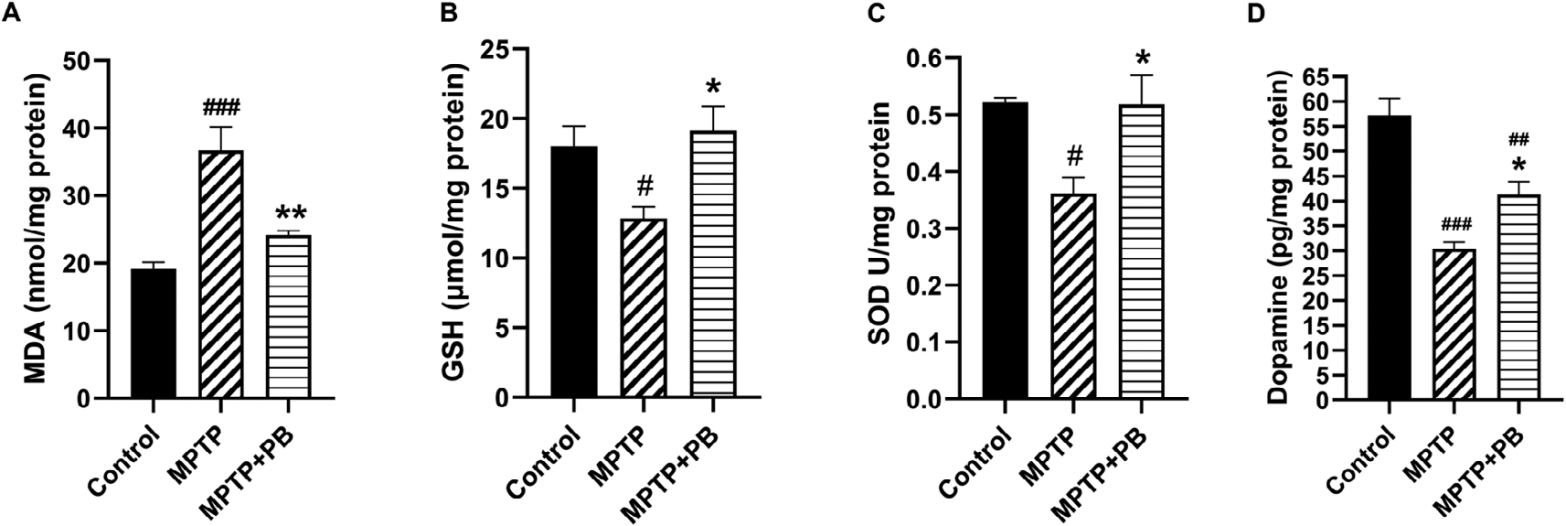
PB treatment attenuated lipid peroxidation as revealed by MDA assay (A) and MPTP-induced oxidative stress by upregulation of antioxidative enzymes (B, C). Alteration of DA levels was shown in D. Results were displayed as mean±SD (n=6), one-way ANOVA. ^#^p<0.05, ^##^p<0.01, and ^###^p<0.001 *versus* control group; *p<0.05, and **p<0.01 *versus* MPTP group.

Injection of MPTP caused an accumulation of MDA content as an indicator for lipid peroxidation due to formation of free radicals. However, PB-treated animals revealed a capacity to reduce the MDA content compared to animals in the MPTP group (Control: 19.16±2.44 *versus* MPTP: 36.69±8.52 *versus* MPTP+PB: 24.20±1.50; Figure 3A). The reduction of MDA level in PB-treated animals may be due to an increase of antioxidant enzymes activities. Our data demonstrate that GSH level in the PB-treated group significantly increased in comparison to the MPTP group (Control: 18.06±3.44 *versus* MPTP: 12.85±2.05 *versus* MPTP+PB: 19.13±4.23; Figure 3B). Additionally, PB also elevated the level of SOD. Seven days after MPTP injection, SOD level was markedly increased in the PB-treated group when compared to the MPTP group (Control: 0.52±0.02 *versus* MPTP: 0.36±0.07 *versus* MPTP+PB: 0.51±0.12, Figure 3C).

The improvement of motor coordination in PB-treated animals after exposure to the dopaminergic neurotoxin may be linked to an increase in DA levels. Therefore, alterations of DA were assessed using an ELISA kit on day 7 after MPTP injection. Animals injected with neurotoxin (MPTP) demonstrated markedly reduced DA levels when compared to the control group (Control: 57.18±8.43 *versus* MPTP: 30.34±3.34 *versus* MPTP+PB: 41.30±6.43; Figure 3D). In this study, PB treatment significantly attenuated the reduction of DA when compared to the MPTP group. These results revealed that PB treatment inhibits MPTP-induced oxidative stress via upregulation of antioxidative enzymes; moreover, PB counteracted the loss of DA which correlated with an improvement of motor coordination.

### PB prevents MPTP-induced neuronal loss in the hippocampus

**Figure 4.**
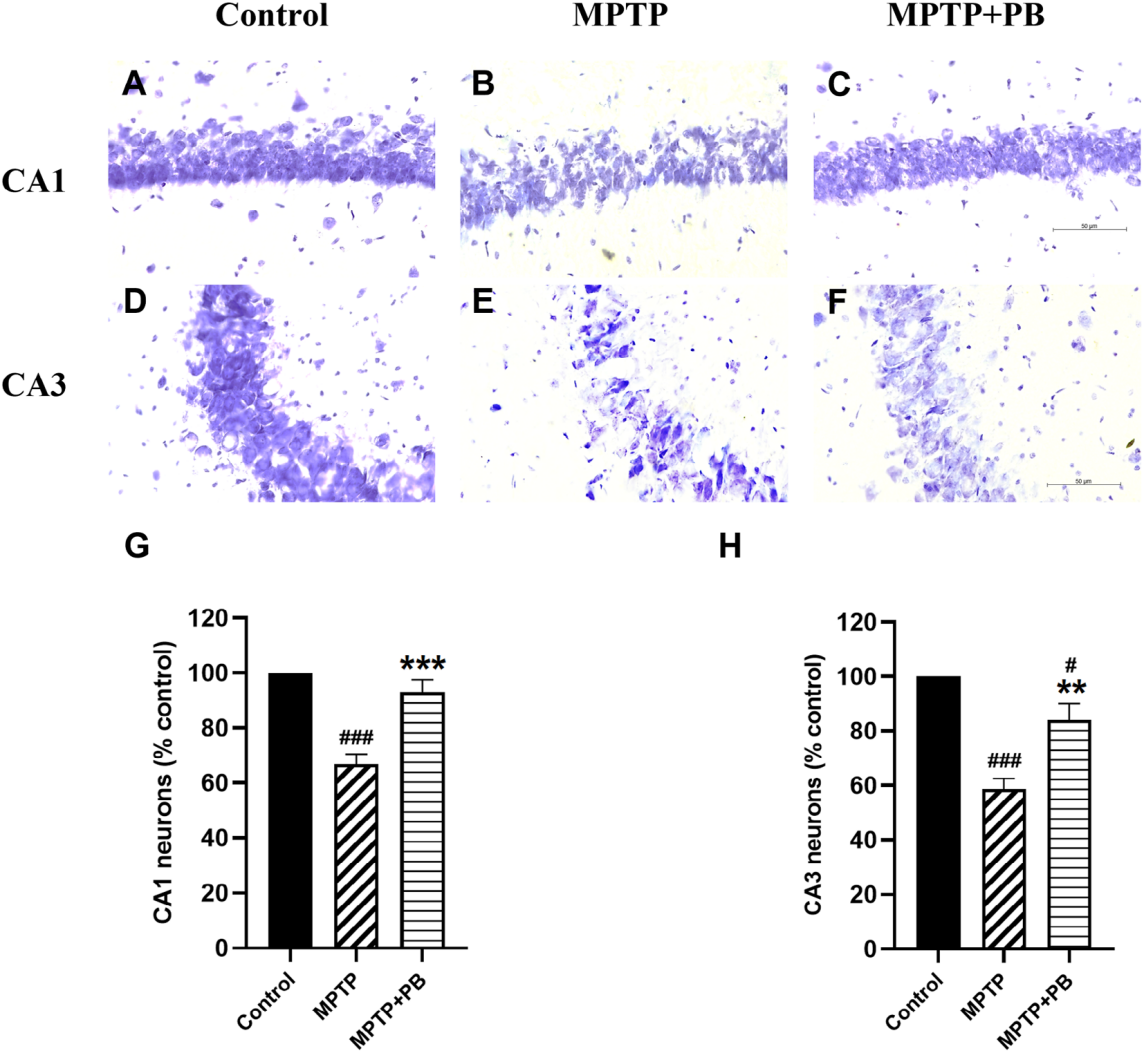
Nissl staining of rat CA1 (A-C), and CA3 (D-F) subfields at 40× magnification. G showed quantification of CA1 neurons and H of CA3 neurons as percentage of control. Data were reported as mean±SEM (n=6), one-way ANOVA. ^#^p<0.05, and ^###^p<0.001 *versus* control group; ^**^p<0.01, and ^***^p<0.001 *versus* MPTP group. Bar=50 μm.

Injection of MPTP caused neuronal apoptosis in the hippocampus and resulted in cognitive impairment in PD. ^26^ In this study, Nissl staining was performed to detect hippocampal neuron degeneration after i.p. injection of MPTP. Overall neuron numbers from all experimental groups were counted at the same levels along the rostro-caudal axis. Neuronal loss was observed in CA1 and CA3 regions of MPTP-treated animals when compared to control rats ( Figure 4). PB administration significantly attenuated the death of principal neurons in the CA1 subfield (MPTP: 66.90±5.51% *versus* MPTP+PB: 92.98±4.67%; Figure 4G). Similarly, animals in the PB-treated group displayed a greater percentage of CA3 neurons than those in the MPTP only group (MPTP: 58.63±3.92% *versus* MPTP+PB: 84.05±6.03%, Figure 4H). These results illustrated that administration of PB could protect hippocampal neurons from death induced by MPTP.

### Alterations in TH and GDNF expressions

**Figure 5.**
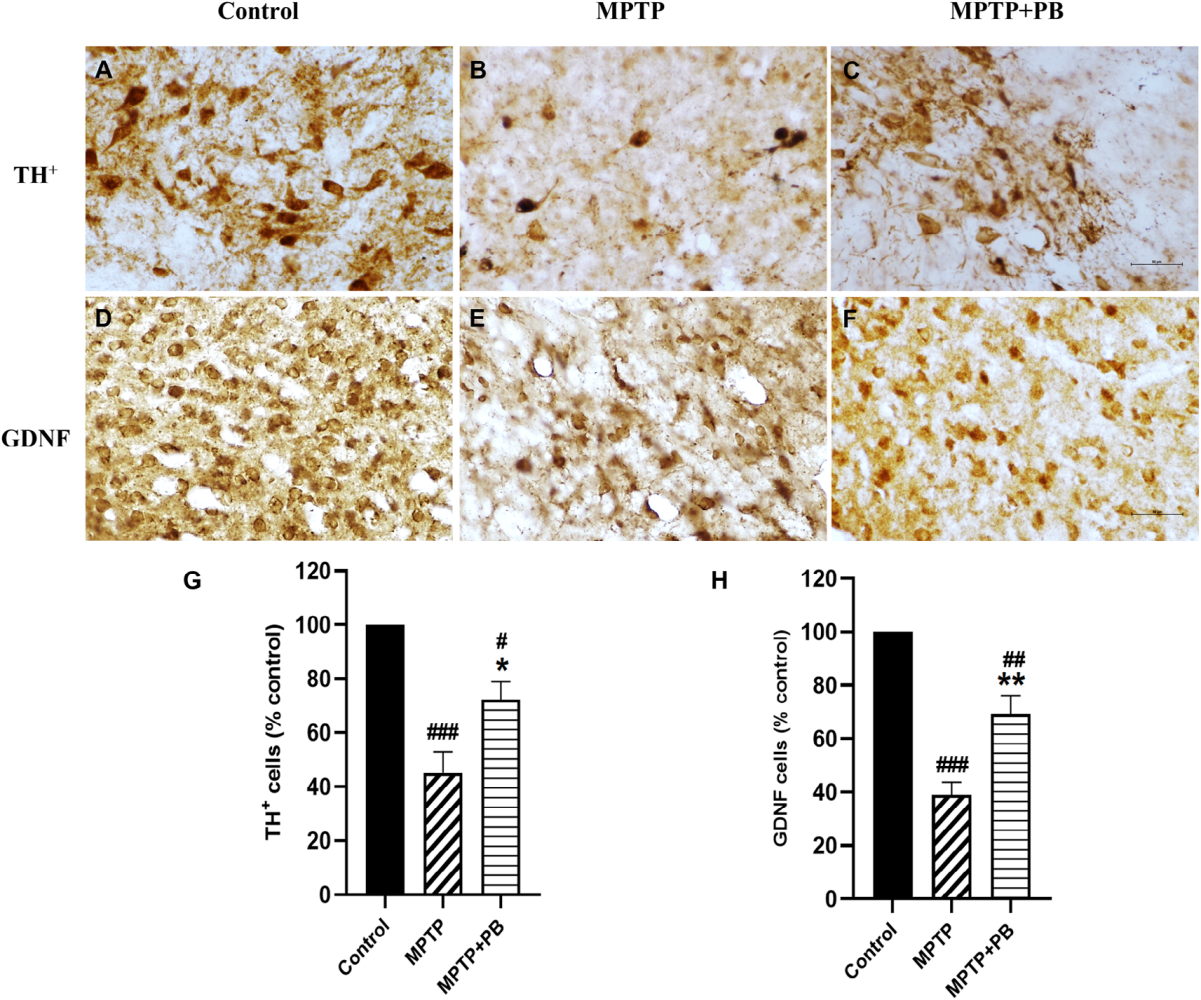
TH staining of SNpc (A-C) presented as number of neurons in percentage of control (G). GDNF-positive immunolabelling in corpus striatum (D-F) were counted and shown in percentage of control as well (H). Images were taken at 40× magnification. mean±SEM (n=6), one-way ANOVA. ^#^p<0.05, ^##^p<0.01, and ^###^p<0.001 vs control group; ^*^p<0.05, and ^**^p<0.01 *versus* MPTP group. Bar=50 μm.

Immunohistochemistry staining of TH was performed to detect neurons containing dopamine in the SNpc. TH is the key enzyme for catecholamine synthesis by converting L-tyrosine to L-DOPA, a precursor for DA. DA insufficiency in the striatum is due to the degeneration of dopaminergic neurons which leads to a decrease in TH activity. ^27^ A previous study found that MPTP decreased TH activity in rodent models. ^28^ Seven days after MPTP injection, TH-positive neurons were dramatically reduced as compared with the control group ( Figure 5A-C). However, there were more TH-positive neurons that survived in MPTP+PB animals (MPTP: 45.15±7.65% *versus* MPTP+PB: 72.22±6.66%, Figure 5G).

We further investigated the expression of GDNF. GDNF has been reported to be a neurotrophic factor of dopaminergic neurons and to protect them from MPTP-induced neuronal apoptosis. ^29^^,^^30^ In this study, PB-treated animals exhibited significantly higher numbers of GDNF-positive immunolabelling in the corpus striatum when compared to animals in the MPTP group ( Figure 5D-F). Quantitative analysis showed a higher percentage in the MPTP+PB group as compared to the MPTP only group (MPTP: 38.93±4.82% *versus* MPTP+PB: 69.22±6.98%, Figure 5H).

## Discussion

PD is characterized by a reduction of dopamine-producing neurons in the SNpc; however, its etiology remains under debate. Recently, the standard treatment for PD has been a dopamine substitution with L-DOPA, but the medications cannot prevent dopaminergic neuron death. ^31^ So far, the high expression of oxidants such as ROS has been considered as the main etiopathology of PD. ^9^^,^^10^ In our previous study, PB as a natural antioxidant agent was shown to attenuate memory impairment and hippocampal neuronal loss, as well as increased astrocytic immunoreactivities of GFAP probably via the activations of antioxidative enzymes including SOD and CAT in chronic stress-induced oxidative stress animal models. ^32^ It is well known that administration of MPTP-induced oxidative stress causes hippocampal neuron loss and depletion of dopaminergic neurons as well as functional impairments. ^33^ The administration of MPTP has been reported to induce locomotor problems in PD animals. ^34^ In the previous investigation, the results demonstrated that MPTP-treated animals exhibited motor function impairments as compared to the naïve control animals. Of note, these deficits improved upon PB treatment seven days after MPTP injection. MPTP plays a crucial role in inducing dopaminergic neuron apoptosis via production of oxidative stress. ^13^ Previous evidence has shown that flavonoids have antioxidant effects that inhibit free radical formation induced by lipid peroxidation. ^35^ In the present study, animals subjected to MPTP for seven days displayed significant activation of lipid peroxidation; a process in which free radicals destroy lipids in cellular membranes as indicated by an increase of MDA levels compared to control animals. MDA levels were decreased in PB-treated animals, and prolonged treatment with PB caused an upregulation of antioxidant enzymes, including GSH and SOD. Previous research has demonstrated that PB attenuates oxidative stress induced by MPTP via the activation of GSH and SOD in the SH-SY5Y cells. ^21^ Our findings confirmed the antioxidant capacity of PB and its neuroprotective effects in neurotoxin-induced neuronal death.

MPTP-caused dopaminergic neuronal cell loss leads to a decrease in dopamine expression in the nigrostriatal system. Previous studies have shown that MPP ^+^-caused mitochondrial dysfunction leads to neuronal apoptosis in the SNpc and in the hippocampus. ^36^^–^^38^ In the present study, PB promoted neuroprotection against neurotoxin induced neuronal apoptosis in the SNpc and in the hippocampus. The animals in the PB-treated group revealed significantly higher numbers of CA1 and CA3 neurons in the hippocampus as well as TH-positive neurons in the SNpc, compared with MPTP treated animals. Moreover, ELISA result showed that PB treatment increased DA levels in the midbrain, which correlates with higher numbers of TH positive neurons as compared to non-treated animals. Hence, higher levels of DA in the nigrostriatal pathway caused an improvement in motor function after MPTP induced PD.

PB prevented neuronal death, possibly via an upregulation of neurotrophic factors. It is well known that GDNF acts as a prominent neurotrophic factor in the DA system and prevents neuronal apoptosis induced by 6-OHDA. ^39^ Moreover, GDNF promotes the survival of dopaminergic neurons after exposure to MPTP. ^40^ Studies have demonstrated that GDNF treatment increased DA release in the striatum. ^41^^,^^42^ High expression of GDNF markedly increases TH-positive neurons in the SNpc, which leads to a reduction in dyskinesia in the MPTP-induced PD. ^30^ In this research, we found that PB treatment could increase the expression of GDNF, as revealed by immunohistochemistry. Previous study has shown the colocalization of GDNF and GFAP-positive astrocytes (astrocytic GDNF), which can increase the striatal neurotransmitters, resulting in a reduction of motor functional deficits. ^40^ Taken together, treatment of PB at a dose of 40 mg/kg could attenuate oxidative stress through an upregulation of antioxidative enzymes and activation of the PI3K/Akt and Erk pathways, which support cellular survival. ^43^^,^^44^

In summary, the present study provides evidence that PB possesses antioxidant activities and neuroprotective effects on MPTP-caused neurodegeneration in PD leads to the improvement of the motor function. However, the limitation of this study is no observation about the effect of PB on depression or cognition related Parkinson’s disease. Our data provide that PB may serve as a therapeutic drug for PD or in neurodegenerative diseases caused by free radical-induced neuronal cell death.

## Data Availability

Figshare: Pinostrobin mitigates neurodegeneration through an up-regulation of antioxidants and GDNF in a rat model of Parkinson’s disease, https://doi.org/10.6084/m9.figshare.23058479.v3. ^45^ This project contains the underlying following data:
• Ladder rung data • MDA detection data • GSH detection data • SOD detection data • Dopamine detection data • Hippocampal cresyl violet staining images • Striatal GDNF immunohistochemistry staining images • Substantia nigra tyrosine hydroxylase immunohistochemistry staining images Figshare: ARRIVE checklist for ‘Pinostrobin mitigates neurodegeneration through an up-regulation of antioxidants and GDNF in a rat model of Parkinson’s disease’, https://doi.org/10.6084/m9.figshare.23058479.v3. ^45^ Data are available under the terms of the Creative Commons Attribution 4.0 International license (CC-BY 4.0).
