# Supplementary material for: Pinostrobin mitigates neurodegeneration through an up-regulation of antioxidants and GDNF in a rat model of Parkinson’s disease
Source: F1000Res. 2023 Oct 27;12:846. Originally published 2023 Jul 18. [Version 2] doi: 10.12688/f1000research.134891.2 (PMC10904945; doi:10.12688/f1000research.134891.2)
Supplement: Peer review history

# Reviewer report: Reviewer response for version 2

**DOI:** 10.5256/f1000research.157966.r242318

**Published:** 2024-02-26

**Authors:** Radha Goel

**Affiliations:** 1 Lloyd Institute of Management & Technology, Greater Noida, India

The authors well written the manuscript  entitled  "Pinostrobin mitigates neurodegeneration through an up-regulation of antioxidants and GDNF in a rat model of Parkinson’s disease"

1. As per my suggestion the authors must provide the histology as mentioned in the manuscript.
2. The discussion must be more elaborate as per the results of the study.

Is the work clearly and accurately presented and does it cite the current literature?

Yes

If applicable, is the statistical analysis and its interpretation appropriate?

Yes

Are all the source data underlying the results available to ensure full reproducibility?

Yes

Is the study design appropriate and is the work technically sound?

Yes

Are the conclusions drawn adequately supported by the results?

Yes

Are sufficient details of methods and analysis provided to allow replication by others?

Yes

Reviewer Expertise:

Neuropharmacology

I confirm that I have read this submission and believe that I have an appropriate level of expertise to confirm that it is of an acceptable scientific standard.

# Reviewer report: Reviewer response for version 2

**DOI:** 10.5256/f1000research.157966.r217483

**Published:** 2023-11-06

**Authors:** Fermin Pacheco-Moises

**Affiliations:** 1 Chemistry, Universidad de Guadalajara, GUadalajara, Jalisco, 44420, Mexico

The paper shows experimental data obtained in an animal model of Parkinson's disease. For this purpose they used pinostrobin, a natural flavonoid extracted from the rhizomes of B. rotunda. Previously, the authors showed that a dose of 40 mg/kg body weight has beneficial effects in promoting remyelination and cell survival in a model of sciatic nerve damage and that this effect is associated with its antioxidant properties. 

 The protective effect of molecules with antioxidant activity in animal models of PD is widely documented in the literature. The authors demonstrate that pinostrobin improves motor coordination and prevents neuronal death in regions of the hippocampus and substantia nigra of animals with symptoms similar to PD and that this effect is related to a decrease in some markers of oxidative stress and with the levels of expression of the glial cell line-derived neurotrophic factor.

 The methods used for the evaluation of oxidative stress markers as well as immunohistochemistry and specific stains are those of standard use in most research laboratories and are adequate. Something that seems important to me is that the authors use a simple but effective method to evaluate motor activity in the animal model. The discussion of the results is brief but analyzes in some detail the phenomena associated with PD that are relevant to the study. In my opinion, the information presented in the article is sufficient to recommend its indexing.

 It would be desirable for the authors to study in their model other phenomena  that may be of interest, such as inflammation and aspects related to mitochondrial enzymatic activity and mitochondrial dynamics itself. In this regard, it would be interesting to evaluate whether pinostrobin has a positive effect on mitochondrial fusion and fission, transport and mitophagy.

 In my opinion, the study contains information that may be valuable to develop a clinical trial with patients in early stages of Parkinson's

Is the work clearly and accurately presented and does it cite the current literature?

Yes

If applicable, is the statistical analysis and its interpretation appropriate?

Yes

Are all the source data underlying the results available to ensure full reproducibility?

Yes

Is the study design appropriate and is the work technically sound?

Yes

Are the conclusions drawn adequately supported by the results?

Yes

Are sufficient details of methods and analysis provided to allow replication by others?

Yes

Reviewer Expertise:

Biochemical markers in neurodegenerative diseases

I confirm that I have read this submission and believe that I have an appropriate level of expertise to confirm that it is of an acceptable scientific standard.

# Reviewer report: Reviewer response for version 1

**DOI:** 10.5256/f1000research.147982.r205415

**Published:** 2023-10-16

**Authors:** Ana C Dutra-Tavares

**Affiliations:** 1 Universidade do Estado do Rio de Janeiro (UERJ), Rio de Janeiro, RJ, Brazil

In the present paper the authors investigated the effects of a flavonoid substance with anti-inflammatory effects on a rodent model of Parkinson’s disease.

 The findings are interesting since they observed neurochemical, morphological and behavioral reversion of alterations associated with this disease by the pinostrobin. Some comments will be made bellow to improve manuscript and provide to the reader more detailed information.

 1 - Parkinson’s disease is characterized by motor impairments but also cognitive decline and psychiatry disorders, such as depression, could be present. Why did the authors focus only on motor dysfunction? Is there evidence that pinostrobin would be only effective on this type of symptom? Is there evidence that pinostrobin could be effective on the other symptoms domains? Although the authors did not investigate that, a more a broader discussion about other possible therapeutic effects and/or limitations to the pinostrobin application on Parkinson’s disease would benefit the reader.

 2 – It is kind of hard to understand how the substantia nigra was used for biochemical assays and to immunohistochemistry. Was substantia nigra from each hemisphere used to different techniques? It would be helpful if the authors provide more detailed information about the dissection.

 3 – The authors present the staining results in percentage from control group. In this sense, the mean value of control group is equal to 100%, but there is a variability among the individuals. Thus, it is always good to present the error bar to show the variability of the data for this group.

Is the work clearly and accurately presented and does it cite the current literature?

Yes

If applicable, is the statistical analysis and its interpretation appropriate?

Yes

Are all the source data underlying the results available to ensure full reproducibility?

No source data required

Is the study design appropriate and is the work technically sound?

Yes

Are the conclusions drawn adequately supported by the results?

Yes

Are sufficient details of methods and analysis provided to allow replication by others?

Partly

Reviewer Expertise:

neurophysiology; neuropsychiatric disorders animal models.

I confirm that I have read this submission and believe that I have an appropriate level of expertise to confirm that it is of an acceptable scientific standard, however I have significant reservations, as outlined above.

# Response

**Published:** 2023-10-17

**Authors:** Sitthisak Thongrong

**Affiliations:** Division of Anatomy, University of Phayao, Mueang Phayao District, Phayao, Thailand

Thank you for your valuable comments that we have followed them for improving the manuscript. Here, we have focused on the effects of pinostrobin in a rat model of Parkinson's disease. 

 1. We have studied that pinostrobin could improve cognitive impairment induced by chronic stress (reference number 32) that is why here we focused only on the motor function. However, this must be our weak point because we did not observe the effect of pinostrobin on cognitive decline and psychiatry disorders so we will put this in the limitation of this study.

 2. We used the right hemisphere for biochemical assays and the left hemisphere for histological studies. The way to dissect the midbrain including substantia nigra was added to the manuscript. 

 3. Your comment is worth for us. We made the normalization that is why the control groups did not show the error bar. However, the F1000research let us show all the raw data that the readers can access them whenever they want.
